# Toward a Romanian Version of The Three-Factor Eating Questionnaire-R21 for Children and Adolescents (CTFEQ-R21): Preliminary Psychometric Analysis and Relation with Body Composition

**DOI:** 10.34763/devperiodmed.20192301.4553

**Published:** 2019-04-08

**Authors:** Mirela Steff, Julien Verney, Marius Marinau, Sergiu Perte, Bruno Pereira, Eleonor Bryant, Vicky Drapeau, Jean-Philippe Chaput, Daniel Courteix, David Thivel

**Affiliations:** 1 Faculty of Geography, Tourism and Sports, Research Centre on Human Performance, University of Oradea, Oradea Romania; 2 Laboratory of the Metabolic Adaptations to Exercise under Physiological and Pathological Conditions (AME2P EA 3533), University of Blaise Pascal in Clermont-Ferrand, Clermont Auvergne University, CRNH-Auvergne, Clermont-Ferrand, France; 3 Biostatistics Unit (DRCI), Clermont-Ferrand University Hospital, Clermont-Ferrand, France; 4 Division of Psychology, Faculty of Social Sciences, University of Bradford, Bradford, West Yorkshire, UK; 5 Department of Physical Education, Faculty of Education, Université Laval, Québec City, QC, Canada; 6 Healthy Active Living and Obesity Research Group, Children’s Hospital of Eastern Ontario Research Institute, Ottawa, Ontario, Canada

**Keywords:** children, eating behaviours, obesity, psychometrie properties, validation

## Abstract

**Purpose:**

The aim of this study was to develop and validate a Romanian version of the Three-Factor Eating Questionnaire-R21 for children and adolescents CTFEQ-R21 and to assess its psychometrie properties and factor structure. Associations between the present version of the CTFEQ-R21 and anthropometric measures as well as body composition were also examined.

**Material and methods:**

153 children and adolescents (68 boys and 95 girls; 10.8±3.5 years) took part in this study (BMI of 17.7±3. 1kg/m^2^). The participants were fitst interviewed to ascertain their understanding of the CTFEQ-R21 and were then asked to self-complete the questionnaire. Height and weight were measured and body composition assessed using bio impedance analyzers (Tanita MC 780).

**Results:**

The CTFEQ-R21 showed satisfactory interna/ consistency (Cronbach's a=0.78). Cronbach's alpha coefficients were 0.55 for CR (cognitive restriction), 0.75 for UE (Uncontrolled Eating), and 0.76 for EE (Emotional Eating) separately. UE and EE were found to be significantly correlated (r=0.54, p<0.05). The three factors explained 43% of the toto/ variance. Correlation between CR, UE and EE with body weight, BMI and FFM were significant but low to moderate, with coefficients ranging from 0.20 to 0.37. The higher the CR, UE and EE tertiles, the higher the weight, fat mass (kg) and fat-free mass values.

**Conclusions:**

According to the psychometrie analysis of the questionnaire, the version of the CTFEQ-R21 proposed here is a satisfactory tool to assess eating behaviors in the Romanian child population that remains to be further developed.

## Introduction

The continuous worldwide progression of overweight and obesity in pediatric populations is becoming one of the most critical global public health concerns [1,2]. While most westernized countries have been working on public health strategies aimed at promoting a healthy lifestyle, developing countries are currently facing the alarming progression of physical inactivity, sedentary behaviors and unhealthy eating behaviors. In 2013 the World Health Organization (WHO) and the European Childhood Obesity Surveillance Initiative (ECOSI) reported an overweight/obesity prevalence of 26.8% (11.6% for obesity) in 8-year-old children from Romania (based on the WHO reference curves) [3], which placed Romania among the worst countries in the world in term of excess weight [4]. More recently, Emandi and collaborators reported that almost one in four Romanian children aged 6-19 years, were overweight or obese between 2006 and 2015 [5].

Although valid universal tools are available to objectively and properly measure physical activity (e.g., accelerometers), valid methods to assess the cognitive and behavioral dimensions of eating behaviors, as well as attitudes towards food, remain to be developed in Romania. The Three Factor Eating Questionnaire (TFEQ) [6] has been specifically developed to assess individuals’ eating behaviors, and its accuracy has been shown in different populations [7, 8, 9, 10]. Recently, a short form of the TFEQ composed of 21 items (CTFEQ-R21) has been developed to measure the cognitive, behavioral and emotional dimensions of eating behaviors [10, 11, 12]. These three factors refer to “Cognitive Restraint” (CR), i.e. conscious effort to control what an individual ingests in order to maintain or decrease body weight, “Uncontrolled Eating” (UE), i.e. a tendency to over-consume food in response to the loss of control over the food itself; and “Emotional Eating” (EE), i.e. the need to overeat when individuals do not manage to cope with emotionally negative events and mood. This shorter TFEQ form has been widely used among adults [11, 13, 14, 15], with a growing interest in children and adolescents [9, 10].

Some studies have been interested in the relationship between weight status or body composition with eating behaviors in children and adolescents [6, 9, 10, 11, 12, 16, 17]. Using the original 51 -item version of the TFEQ, it has been shown that cognitive or restrained eating was positivity associated with children and adolescents’ body mass index (BMI) and body mass [6, 18]; however, the relationships between adiposity and emotional or external/ uncontrolled eating are unclear [19]. Recently, Bryant et al. developed and validated a pediatric version of the TFEQ (CTFEQ-R17), showing that CR was associated with a higher body weight, BMI and BMI percentile and that high UE and EE scores were related to a preference for high fat savoury and high fat sweet foods in 12-year-old children and adolescents [9]. Martin-Garcia and collaborators also found that CR was strongly and positively related to body composition in Spanish children and adolescents using an adapted Spanish version of the TFEQ-R based on 21 times (TFEQ-R21) [10].

## Aim

While there is a growing interest in the prevention and treatment of pediatric overweight and obesity in Romania, there is also a need for adapted and validated tools to properly assess children and adolescents’ eating behaviors. The purpose of the present study was to validate a Romanian version of the TFEQ for children and adolescents and to analyze its psychometric properties and factor structure. A second aim was to explore the relationships between Romanian children’s and adolescents’ eating behaviors and their body composition.

## Material and methods

### Study population

A total of 153 children and adolescents (68 boys and 95 girls) took part in this study, with a mean age of 10.8 ± 3.5 years (age ranged from 6 to 16 years) and a mean BMI of *17.7±3.1* kg/m2. To be included in the study, children and adolescents had to be free of any history of psychological or eating disorders. None of the children and adolescents was following a diet, nor were they taking any medication that could interfere with the results. The participants were recruited among primary and secondary schools in Oradea, Romania. Both the participants and their legal representatives received information sheets and signed consent forms. The study was conducted in accordance with the Declaration of Helsinki of 1975 regarding the ethical principles for human research and after consideration by the appropriate local authorities (University of Oradea ethical commission).

### Validation process of the CTFEQ-R21C

The Romanian version of the CTFEQ-R21 (see Appendix 1 ) was obtained after the following process (as described by [10]: i) translation and back translation procedure by two independent Romanian native speakers fluent in English of the CTFEQ [9]; ii) back translation review and harmonization between the new translation and the source version; and iii) review of the translation by an expert in pediatric nutrition and adaptation of the vocabulary when necessary. The final version of the questionnaire was composed of the same number of items (21 questions), the 4-point response scale for answering from questions 1 to 20, and an 8-point response scale for item 21. The codification and treatment proposed by Cappelleri et al. & Bryant et al. to obtain the three factors (UE, CR, and EE) were also retained [9, 11].

### Experimental design

The children and adolescents enrolled in the study were asked to join the university facilities where they received verbal and written instructions on how to properly complete the questionnaire. They answered the questions by themselves and a member of the investigation team was present to help them when necessary, especially the youngest participants. The questionnaire took about 10 to 15 minutes to complete. Anthropometric and body composition measurements were also taken using standardized procedures.

**Appendix 1:** The Romanian version of the 21-item Three Factor Eating Questionnaire for children and adolescents (CTFEQ-R21C).

1.	Mânânc por<b>ii mici la masǎ pentru a ma ajuta sǎ-mi controlez greutatea
	(1) în totalitate de acord / (2) Probabil adevǎrat / (3) Probabil fais / (4) an totalitate fals
2.	încep sǎ mânânc atunci când mǎ simt îngrijorat/ǎ.
	(1) în totalitate de acord / (2) Probabil adevǎrat / (3) Probabil fais / (4) în totalitate fais
3.	Uneori, când încep sǎ mânânc mi se pare CD nu ma pot opri.
	(1) în totalitate de acord / (2) Probabil adevǎrat / (3) Probabil fais / (4) în totalitate fais
4.	Când mǎ sim trist/ǎ, de obicei mânânc mult.
	(1) în totalitate de acord / (2) Probabil adevǎrat / (3) Probabil fais / (4) an totalitate fais
5.	Nu mânânc anumite tipuri de mâncare pentru cǎ pot sa mǎ îngraş.
	(1) în totalitate de acord / (2) Probabil adevǎrat / (3) Probabil fais / (4) an totalitate fals
6.	Când sunt lângâ cineva care mânâncâ, şi mie îmi vine poftǎ de mâncare.
	(1) în totalitate de acord / (2) Probabil adevärat / (3) Probabil fais / (4) în totalitate fals
7.	Când sunt nervos simt nevoia sa mânânc.
	(1) în totalitate de acord / (2) Probabil adevǎrat / (3) Probabil fais / (4) an totalitate fals
8.	De obicei îmi este atât de foame încât simt cǎ aş putea sǎ mânânc mult fǎrǎ sǎ mǎ simt plin/ǎ.
	(1) în totalitate de acord / (2) Probabil adevǎrat / (3) Probabil fals / (4) în totalitate fals
9.	Când îmi este foame simt cǎ trebuie sǎ mânânc tot ce am în farfurie fǎrǎ sǎ mǎ opresc
	(1) în totalitate de acord / (2) Probabil adevǎrat / (3) Probabil fals / (4) în totalitate fals
10.	Când mǎ simt singur/ǎ, mǎ consolez mâncând.
	(1) în totalitate de acord / (2) Probabil adevǎrat / (3) Probabil fais / (4) în totalitate fals
11.	în timpul meselor,motivul pentru care mânânc mai pu<b>in este pentru a nu mǎ ingrǎsa.
	(1) în totalitate de acord / (2) Probabil adevǎrat / (3) Probabil fais / (4) în totalitate fals
12.	Atunci când vǎd sau simt alimentul meu preferat, nu pot sǎ nu îl mânânc chiar dacǎ sunt satul/ǎ.
	(1) în totalitate de acord / (2) Probabil adevǎrat / (3) Probabil fals / (4) în totalitate fals
13.	întotdeauna mi-e destul de foame pentru a mânca la orice orǎ.
	(1) în totalitate de acord / (2) Probabil adevǎrat / (3) Probabil fals / (4) an totalitate fals
14.	Dacǎ mǎ simt nervos/nervoasä, încerc sǎ mǎ calmez mâncând.
	(1) în totalitate de acord / (2) Probabil adevǎrat / (3) Probabil fals / (4) în totalitate fals
15.	Când vǎd ceva ce pare delicios, adesea mi se face foame şi FMI vine sǎ-l mânânc pe moment.
	(1) în totalitate de acord / (2) Probabil adevǎrat / (3) Probabil fals / (4) în totalitate fals
16.	Când mǎ simt eu adevǎrat furios/furioasǎ, vreau sǎ mânânc.
	(1) în totalitate de acord / (2) Probabil adevǎrat / (3) Probabil fals / (4) în totalitate fals
17.	De câte ori evi<b>i sǎ mânânei sau sǎ cumperi alimentele preferate ?
	(1) Aproape niciodatǎ / (2) Câteodatǎ / (3) De obicei / (4) Aproape tot timpul
18.	De câte ori mânânci mai pu<b>in decât ai vrut la o masǎ?
	(1) Aproape niciodatǎ / (2) Câteodatǎ / (3) De obicei / (4) Aproape tot timpul
19.	Ti se întâmplâ sǎ mânânei mult chiar dacǎ nu îti este foame?
	(1) Niciodatǎ / (2) Nu foarte des / (3) Câteodatǎ / (4) Cel pu<b>in odatǎ pe sǎptǎmǎnǎ
20.	Cât de des î<b>i este foame?
	(1) Numai înainte de masâ / (2) Uneori între mese / (3) De multe ori între mese / (4) Aproape tot timpul
21.	Ce tip de mâncâcios eşti pe o scarǎ de 1 la 8? Unde 1 semnificǎ : „Mânânc numai ce vreau şi când vreau" şi 8 semnificǎ: „Sunt atent la ce mânânc pentru a-mi controla greutatea". *12345678*

### Anthropometric and body composition measurements

A digital scale was used to measure body mass to the nearest 0.1 kg, and standing height was assessed barefoot, to the nearest 0.1 cm by using a wall-mounted stadiometer. BMI was calculated as body mass (kg) divided by height squared (m2). Body composition was assessed by bioelectrical impedance using a Anita MC-780 multi-frequency, segmental body composition analyzer. This consisted of a stand-alone unit which the participant had to step on barefoot (standard mode). Information about the participant (age, sex, and height) was entered by the experimenter. Once body mass had been assessed by the Tanita scale, the participant had to take grips in both hands (alongside his/her body) during the impedance measure (hand to foot). A full segmental analysis was performed in less than 20 s. Segmental fat mass and fat-free mass values were indicated at the end of the analysis on the digital screen (trunk, left and right arms and legs); as well as total body fat, fat-free mass and water. This newly developed technology has been recently validated in adults of various physical activity levels [20] and children of various adiposity levels [21], showing a good level of validity.

### Statistical analysis

All statistical analyses were performed using Stata software (Version 13, StataCorp, College Station, US) and the level of significance set at p<0.05. Analyses performed in this study were those usually used in studies aimed at validating scales. In addition to descriptive statistics, the following psychometric properties of the questionnaire were explored. Acceptability: Data quality was considered satisfactory if more than 95% of the scale data were fully computable. Score range, closeness of mean to median, floor and ceiling effects (accepted maximum for both: 15%), and skewness of score distributions (limits: -1 to +1) were also analyzed. An *exploratory factor analysis* (principal components analysis method with varimax rotation) was carried out to determine the scale structure. The number of factors was chosen according to usual recommendations: Kaiser criteria, plot of eigenvalues and part of variance expressed by principal components. The Kaiser-Meyer-Olkin test for sample adequacy was applied. *Internal consistency* was determined through Cron bach's alpha coefficient (minimum accepted value: 0.70); the item homogeneity coefficient (criterion value: > 0.30), and the item-total correlation corrected for overlap (criterion value: >0.30. *Internal validity* was determined by correlation coefficients between the domains composing the scale (standard, p=0.30-0.70). Regarding convergent validity, a high association (Spearman rank correlation coefficient, p>0.50) was hypothesized between the scale scores and other quantitative measures (weight, BMI, fat-free mass and fat mass). Then, each dimension of the scale score was categorized in 3 modalities according to statistical distribution (< first quartile, first-third quartile, > third quartile; respectively named Tertile 1, tertile 2 and Tertile 3 in the result section). The comparisons between groups for weight, BMI, fat-free mass and fat mass were performed by ANOVA or non-parametric Kruskal-Wallis test, followed by a post-hoc test for multiple comparisons (Tukey-Kramer post ANOVA and Dunn after the Kruskal-Wallis test). Relationships between quantitative variables were assessed using correlation coefficients and were represented graphically with a color-coded heatmap. Since we missed to observe any gender differences, the data were analyzed together and all the analyses were adjusted for age and gender.

## Results

### Internal consistency analysis

In this study, the Cronbach’s alpha coefficient for the whole questionnaire was 0.78. When analyzed separately, the Cronbach’s alpha coefficients were 0.55 for CR, 0.75 for UE, and 0.76 for EE. Only the correlation between UE and EE was found to be statistically significant, with a correlation coefficient of 0.54 (p<0.001). For the relation between CR and the other eating behaviors, the correlation coefficients were low (0.07 and 0.08 for UE and EE, respectively).

**Fig. 1:**
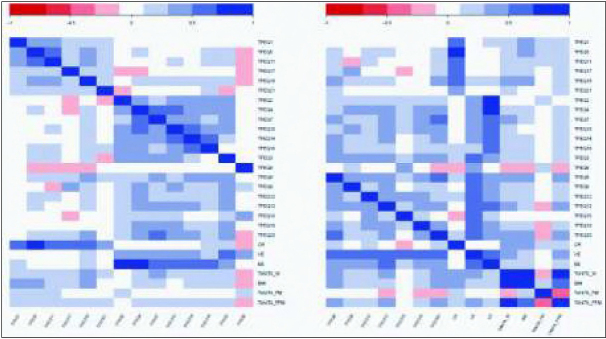
Heatmap representation of the correlations between items of the CTFEQ, between each item and each dimension (EE, CR, UE) and between each item and dimension, and anthropometric and body composition variables. The darkest is the box and the higher is the correlation. CR: Controlled eating; EE: Emotional Eating; UE: Uncontrolled eating; Tanita_W: body weight; BMI: Body Mass Index; Tanita FM: Fat Mass (%); Tanita_FFM: Fat Free Mass (kg).

As illustrated in the color-coded heatmap, correlations between items assigned to a determined scale were moderate and correlations with items of the other two scales remained low, even if the relationships between UE and EE items were stronger (the correlation coefficients range scale is detailed on the heatmap fig. 1).

### Convergent validity - relationships between CTFEQ-R21 and body composition

These results were confirmed by an exploratory factor analysis. According to the maximum likelihood analysis with Varimax rotation performed, the original structure of the CTFEQ-R21 appears to be replicated in this study. The principal components analysis identified three factors with an eigenvalue above 1. These three factors explained 43% of the total variance (tab. I). The factor analysis highlighted that each item loaded positively to one of the three factors that corresponded to the same factors of the original questionnaire (CTFEQ-R21). The Kaiser Meyer Olkin index was 0.75.

**Table I:** Factor loading of the CTFEQ-R21 items and communalities.

Item	Item content	Uncontrolled eating	Emotional eating	Cognitive restraint
13	I'm always hungry enough to eat at any time.	0.31		
3	Sometimes when 1 start eating, 1 just can't seem to stop.	0.28		
15	When 1 see something that looks very delicious, I often get so hungry that I have to eat right away.	0.15		
8	I often get so hungry that my stomach feels like a bottomless pit.	0.31		
6	Being with someone who is eating, often makes me want to also eat.	0.05		
20	How often do you feel hungry?	0.31		
9	I'm always so hungry that it's hard for me to stop eating before finishing all of the food on my plate.	0.22		
12	When I smell a sizzling steak or see a juicy piece of meat, I find it very difficult to keep from eating even if I've just finished a meal.	0.25		
19	Do you go on eating binges even though you're not hungry?	0.24		
16	When I feel depressed, I want to eat.		-0.48	
2	I start to eat when I feel anxious.		-0.26	
10	When I feel lonely, I console myself by eating.		-0.20	
4	When I feel sad, I often eat too much.		-0.26	
14	If I feel nervous, I try to calm down by eating.		-0.37	
7	When I feel tense, I often feel I need to eat.		-0.08	
11	I hold back on how much I eat at meals on purpose to keep from gaining weight.			0.49
1	I take small portions on purpose to control my weight.			0.37
5	I don't eat some foods because they make me fat.			0.49
21	On a scale from 1 to 8, where 1 means no restraint in eating (eating whatever you want, whenever you want it) and 8 means total restraint (constantly limiting food intake and never "giving in"), what number would you give yourself?			0.28
17	When do you avoid "stocking up" on tempting foods?			0.17
18	How likely are you to make an effort to eat less than you want?			0.41
Explained variance		23.39	7.97	11.41
Accumulated variance		23.39	42.77	34.80

First, correlation coefficients were calculated between CTFEQ-R21 dimensions and parameters related to body composition. Values of coefficients for body weight, BMI and FFM were low to moderate (ranged from 0.20 to 0.37), despite significant p-values (tab. I). No significant correlations were found between each CTFEQ-R21 dimension and fat mass, but there were ones for body weight, BMI and FFM (see tab. II).

**Table II:** Correlations between the three eating behaviors from the CTFEQ-R21C and body composition indicators.

	Body Weight (kg)	BMI (kg/m^2^)	Fat Mass (%)	FFM (kg)
Cognitive restraint	0.34*	0.38*	0.14	0.29*
Uncontrolled eating	0.37*	0.31*	-0.08	0.34*
Emotional eating	0.21*	0.20*	0.04	0.21*

BMI: Body Mass index; FFM: Fat-Free Mass; *p<0.05

These results were confirmed by testing comparisons between body composition indicators by tertile of eating behavior scores (tab. III). No association was found between fat mass (%) and the three eating behaviors. However, the higher the CTFEQ-R21 factor tertile, the higher the weight, fat mass (kg) and fat-free mass values.

**Table III:** Mean body composition values of participants stratified by tertile of eating behavior.

Cognitive Restraint	Tertile 1 (Low)		Tertile 2 (Intermediate)		Tertile 3 (High)		ANOVA
	*Mean*	*SD*	*Mean*	*SD*	*Mean*	*SD*	
Body Weight (kg)	37.8	17.3	38.9	15.6	51.6	17.8	**T3>T1/T2 **
FM (kg)	8.1	9.5	7.8	3.7	11.3	5.3	***T3>T1/T2 **
FM (%)	19.3	4.7	20.2	5.1	22.0	6.3	NS
FFM (kg)	29.5	14.6	29.5	12.1	38.2	13.5	*T3>T1/T2*
**Uncontrolled Eating**	**Tertile 1 (Low)**		**Tertile 2 (Intermediate)**		**Tertile 3 (High)**		**ANOVA**
	***Mean***	***SD***	***Mean***	***SD***	***Mean***	***SD***	
Body Weight (kg)	34.3	12.7	38.9	17.1	51.4	17.8	***T3>T1/T2 **
FM (kg)	9.1	11.5	7.9	4.2	9.9	4.6	**T2<T1/T3 **
FM (%)	21.1	5.5	20.7	4.8	19.7	6.1	NS
FFM (kg)	26.6	11.3	29.4	13.2	36.7	13.8	***T3>T1/T2***/TKT2*
**Emotional Eating**	**Tertile 1 (Low)**		**Tertile 2 (Intermediate)**		**Tertile 3 (High)**		**ANOVA**
	***Mean***	***SD***	***Mean***	***SD***	***Mean***	***SD***	
Body Weight (kg)	37.5	16.3	48.8	20.2	45.7	15.9	**T1<T2/T3 **
FM (kg)	8.4	8.3	9.1	4.8	9.7	4.4	*T1<T3*
FM (%)	20.5	4.7	19.3	6.5	21.2	5.5	NS
FFM (kg)	28.7	13.1	36.8	15.8	34.2	12.3	**TKT2/T3**

BMI: Body Mass Index; FM: Fat Mass; FFM: Fat-Free Mass; SD: Standard Deviation; ANOVA: Analysis of Variance; T1: Tertile 1; T2: Tertile 2; T3: Tertile 3. *p<0.05; **p<0.01; ***p<0.001.

## Discussion

This study aimed to test the psychometric properties of a Romanian version of the CTFEQ-R21 to assess eating behaviors in children and adolescents (CTFEQ-R21). This Romanian version of the CTFEQ-21 provides a satisfactory assessment of childrens and adolescents’ eating behaviors, with an internal-consistency Cronbach’s alpha coefficient of 0.78. When analyzed separately, CR, EE and UE showed coefficients of 0.55, 0.76 and 0.75, respectively.

These results are in line with those of Bryant et al. who proposed the adapted version of the TFEQ for children and adolescents showing an internal consistency coefficient of 0.81 [9]. In their study, Martin-Garcia and collaborators proposed a Spanish version of this CTFEQ-R21 among 8- to 17-year- old youth and also found a Cronbach’s alpha coefficient of 0.73 [10]. Similarly to the present work, both the English [9] and Spanish versions [10] of this CTFEQ-R21 found lower internal consistency for the CR dimension compared with UE and EE. As illustrated by the heatmap model, the results showed moderate inter-item correlations, which also supports the available literature [9, 10]. While both the English and Spanish versions of the questionnaire found significant correlations between UE and both EE and CR [9, 10], the results observed in the present study showed a significant correlation only between UE and EE, with CR showing low correlations with UE and EE. In the present study, the three factors explained 43% of the total variance, although this remains moderate compared to the 51.6% variance observed in the English version of the CTFEQ-R21 [9]. Nevertheless, this is better than the 34.4% variance obtained in the study from Martin-Garcia et al. [10].

This Romanian version of the CTFEQ-R21 showed moderate correlations between each dimension of the questionnaire (CR, UE, EE) and body weight, BMI and FFM. However, fat mass was not correlated with CR, UE, or EE. Although Bryant and colleagues also observed significant positive correlations between CR, body weight and BMI (particularly in girls) in a similar population, UE and EE were not related to anthropometric measures in their work [9]. Previously published studies also found significant correlations between CR and anthropometric values [7, 8, 10]. In addition, the present results are in line with other evidence showing significant correlations between body weight/BMI and EE [22], UE [10, 23] or both [24, 25].

In the present study, we also divided data according to tertiles of CTFEQ-R21 eating behavior traits that discriminate against children and adolescents showing the lowest, intermediate and highest values for each of the three dimensions. Accordingly, higher body weight, BMI, fat-free mass and fat mass (kg only) were observed with higher tertiles of eating behaviors. In their work, Martin-Garcia et al. also analyzed their results according to tertiles and found similar results for CR only [10]. According to the later study, children and adolescents in the lower UE fertile showed a significantly higher body weight with no difference for BMI and total fat mass. However, they did not find any difference between anthropometric and body composition measures between the tertiles of EE scores [10]. Such divergent results could be explained by the higher heterogeneity of their sample in terms of weight status with a large majority of overweight/obese participants (51 overweight, 83 obese and 54 lean) [10].

Although this work is the first to propose a version of the CTFEQ-R21 for Romanian children, its results must be interpreted in the light of some limitations. First, the composition of our sample must be considered. The present sample is mainly composed of normal-weight children and adolescents, so further investigations should be conducted among overweight and obese ones. Moreover, the sample presents quite a broad age range, which has to be considered when interpreting our results. The unequal proportion of boys and girls is certainly one of the main limitations of our sample. Indeed, we did not observe gender differences during our analysis, which is certainly due to this disproportionate number of boys and girls. This is of particular importance, since the literature clearly points out differences between boys and girls when it comes to eating behaviors, with girls usually showing significantly higher average results in each of such scale dimensions. Nevertheless, even if our analyses had been controlled for age and sex, it would have been more precise to also evaluate the participants’ biological maturation using Tanner stages. The use of an impedance analyzer to assess body composition might also be considered as a limitation, since more accurate methods, such as Dual-Energy X-ray absorptiometry (DXA) could be used, as in the study of Martin-Garcia et al. [10]. However, the Tanita MC780 BIA analyzer used in the present study has been shown to provide accurate measurements of body composition in children and adolescents [21].

## Conclusion

The proposed Romanian version of the CTFEQ-R21 for children and adolescents shows satisfactory psychometric properties and internal consistency, with anthropometric values, as well as FM and FFM, being correlated with the three eating behavior traits that compose the whole questionnaire. According to the present results, this first version of the Romanian CTFEQ-R21 proposes an interesting tool that needs to be further developed and improved. Further studies enrolling a larger sample should be conducted to specifically question the validity of this questionnaire among overweight and obese Romanian children and adolescents. Other international tools used to assess eating behaviors should be validated in developing countries that are today facing the progression of pediatric obesity which require effective public health strategies.
